# CXCR2^+^ MDSCs promote breast cancer progression by inducing EMT and activated T cell exhaustion

**DOI:** 10.18632/oncotarget.23020

**Published:** 2017-12-07

**Authors:** Ha Zhu, Yan Gu, Yiquan Xue, Ming Yuan, Xuetao Cao, Qiuyan Liu

**Affiliations:** ^1^ National Key Laboratory of Medical Immunology and Institute of Immunology, Second Military Medical University, Shanghai 200433, China

**Keywords:** CXCR2^+^ MDSCs, breast cancer, tumor metastasis, EMT, T cells exhaustion

## Abstract

Although myeloid-derived suppressor cells (MDSCs) have been demonstrated to contribute to tumor initiation, progression and metastasis, however, which MDSC subsets are preferentially expanded and activated, and what’s the key molecular mechanism responsible for specific MDSC subsets in promoting tumor progression need to be fully addressed. Here we identify that Ly6G^mi^Ly6C^lo^CD11b^+^CXCR2^+^ subpopulation (named CXCR2^+^ MDSCs) are predominately expanded and recruited in systemic and local tumor microenvironment during breast cancer progression and metastasis. The proportion of CXCR2^+^ MDSCs is inversely correlated with the infiltration of CD4^+^ or CD8^+^ T cells. Besides, CXCR2^+^ MDSCs promote breast cancer growth and metastasis to lung and/or lymph node *in vivo*. Furthermore, CXCR2^+^ MDSCs induce epithelial mesenchymal transition (EMT) of breast cancer cells via IL-6. Moreover, CXCR2^+^ MDSCs upregulate the expression of immunosuppressive molecules programmed cell death protein 1(PD1), PD1 ligand 1 (PDL1), lymphocyte activation gene 3 protein (LAG3), cytotoxic T lymphocyte antigen 4 (CTLA4), and T cell immunoglobulin domain and mucin domain protein 3 (TIM3) on CD4^+^ or CD8^+^ T cells, and induce exhaustion of the activated T cells partially via IFN-γ. These results demonstrate that CXCR2^+^ MDSCs accelerate breast cancer progression via directly inducing cancer cell EMT and indirectly promoting T cell exhaustion, suggesting that CXCR2^+^ MDSCs may be a potential therapeutic target of breast cancer.

## INTRODUCTION

Myeloid-derived suppressor cells (MDSCs) represent a heterogeneous population of immature myeloid cells which dramatically expand in spleens, peripheral blood or tumor tissues of tumor-bearing hosts, contribute to immune suppression, and adversely associate with patient survival and resistance to various therapies [1]. MDSCs can suppress both innate and adaptive immune responses resulting in promoting tumor angiogenesis, cell invasion and metastasis. MDSCs inhibit innate immune system mostly via secretion of IL-10 and TGF-β, which can drive macrophages to an immunosuppressive M2 phenotype, and negatively affect natural killer cells maturation and dendritic cells (DCs) function [2–4]. The suppressive effect of MDSCs on adaptive immune system is mainly via inhibiting T cells response. MDSCs can deplete the essential amino acids L-arginine and L-cysteine, two amino acids required for T-cell differentiation, and produce reactive oxygen (ROS) and nitrogen species which lead to the loss of T cell receptor (TCR)ζ chain, resulting in decreased T cell differentiation, proliferation and activation [5–7]. Additionally, MDSCs can expand the existing regulatory T cells (Tregs) population and further induce the development of Tregs to suppress T cells immune response [1]. Besides functioning as an immunosuppressive mediator, MDSCs can induce the formation of pre-metastatic niche in lung and bone [8], act as progenitors of osteoclasts and promote osteoclastogenesis [9], adhere to and protect circulating cancer cells from being killed [10]. Given these significant functions of MDSCs in cancer progression, targeting MDSCs as a cancer immunotherapy is under universal research, including preventing the proliferation and development of MDSCs, reducing MDSCs expansion and activation, eliminating MDSCs, and inhibiting the suppressive functions of MDSCs [11]. However, the heterogeneity and the inconsistent phenotypes of MDSCs make the problem more complicated. In mice, MDSCs can be identified by the co-expression of granulocyte marker Gr-1 and macrophage marker CD11b/Mac1 (αM-integrin) on the cell surface. Gr-1 includes two isoforms Ly6C and Ly6G, the differential expression of Ly6C and Ly6G distinguishes monocytic MDSCs (M-MDSCs) from granulocytic MDSCs (G-MDSCs) [12]. The phenotype of M-MDSCs is CD11b^+^Ly6G^−^Ly6C^hi^ and G-MDSCs is CD11b^+^Ly6G^+^Ly6C^lo/−^. Rather than M-MDSCs, G-MDSCs are the subpopulations predominately expand in most cancers [4]. In human, MDSCs express the cell surface markers CD33 and CD11b commonly, express CD14 and CD15 for monocytic and granulocytic MDSCs respectively. Similar to murine MDSCs, human MDSCs lack lineage markers characteristic of other hematopoietic-derived cells. Human G-MDSCs are CD11b^+^CD14^−^CD15^+^HLA^−^DR^low/−^CD33^+^, M-MDSCs are CD11b^+^CD14^+^CD15^−^IL4Rα^+^HLA^−^DR^low^CD33^+^ [13]. Therefore, finding new specific and common markers for MDSCs from both mice and human will benefit the immunotherapy targeting MDSCs.

CXC chemokine receptor 2 (CXCR2) is a member of the G-protein-coupled receptor family. In human, CXCR2 is the receptor for CXCL1, CXCL2, CXCL3, CXCL5, CXCL6, CXCL7 and CXCL8. In mice, CXCR2 interacts with CXCL1, CXCL2, CXCL3, CXCL5 and CXCL7, lack *cxcl6* and *cxcl8* genes in mice [14, 15]. The primary immune function of CXCR2 is to counteract with CXCR4 signals and modulate neutrophils mobilization from bone marrow and migration to inflammatory sites [15, 16]. It has been reported that IL-17a can induce CXCL5 production by liver tumor cells and enhance infiltration of MDSCs into tumor sites in a CXCL5/CXCR2 dependent manner [14]. Similarly, kruppel-like factor KLF4 regulates the recruitment of MDSCs to primary tumor via CXCL5/CXCR2 axis in breast cancer model [17]. CXCR2 has been reported to be mainly expressed by G-MDSCs rather than M-MDSCs [18, 19]. Knock-out of CXCR2 hinders the colitis-associated tumorigenesis through inhibiting CD11b^+^Ly6G^+^CXCR2^+^ MDSCs infiltration into colonic mucosa and tumors. These MDSCs subset can inhibit CD8^+^ T cells cytotoxic activity in colitis-associated tumorigenesis [18]. Additionally, in rhabdomyosarcoma, CXCR2 inhibitor can inhibit CD11b^+^Ly6G^+^CXCR2^+^ MDSCs recruiting to tumor tissues and significantly enforce the anti-programmed cell death protein 1 (anti-PD1) immunotherapy efficacy *in vivo* [19].

Breast cancer is the most common female cancer in China and the second in the USA [20]. Despite advances in early detection and adjuvant therapies, breast cancer is still the most threatening cause of cancer mortality among women. Tumor recurrence and distant metastasis are two major contributors to the death of breast cancer patients [21, 22]. Finding effective targets for breast cancer therapy is still in great need. Recent research has indicated that MDSCs are increased and correlated with type 2 immune responses and poor prognosis in breast cancer patients [23]. Moreover, MDSCs can impair the therapeutic effect. For instance, after receiving doxorubicin-cyclophosphamide chemotherapy, MDSCs in peripheral blood of breast cancer patients will increase and exert immunosuppressive effect [24]. Besides, MDSCs also participate in assisting immune checkpoint blockade in cancer partially [25]. Given the crucial roles of MDSCs in breast cancer patients mentioned above, MDSCs have the potential to be candidate diagnostic markers and therapeutic targets for breast cancer.

In this study, we found that Ly6G^mi^Ly6C^lo^CD11b^+^ CXCR2^+^ subsets (named CXCR2^+^ MDSCs) were predominately expanding and recruiting in systemic and local tumor microenvironment during breast cancer progression. CXCR2^+^ MDSCs could promote breast cancer growth and metastasis to lung and/or lymph node *in vivo*. Furthermore, CXCR2^+^ MDSC subsets induced breast cancer cells epithelial mesenchymal transition (EMT) via IL-6. Moreover, CXCR2^+^ MDSCs upregulate CD4^+^ or CD8^+^ T cells immunosuppressive molecules including PD1, PD1 ligand 1 (PDL1), lymphocyte activation gene 3 protein (LAG3), cytotoxic T lymphocyte antigen 4 (CTLA4), and T cell immunoglobulin domain and mucin domain protein 3 (TIM3) via IFN-γ partially. These results demonstrate that CXCR2^+^ MDSCs can accelerate breast tumor growth and metastasis via directly inducing cancer cells EMT and indirectly promoting T cells exhaustion, suggesting that CXCR2^+^ MDSCs may be a potential target for breast cancer therapy.

## RESULTS

### CXCR2^+^ MDSCs predominantly expanded and accumulated during breast cancer progression

**Figure 1:**
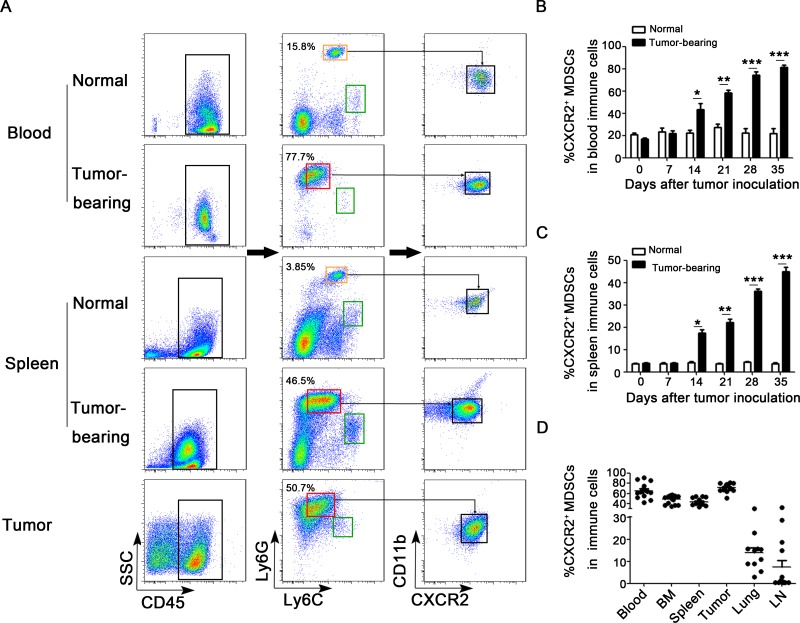
CXCR2^+^ MDSCs are predominately expanded and recruited during breast cancer progression (**A**) Flow cytometric analysis of single cells isolated from peripheral blood, spleens and tumor tissues from tumor-bearing mice as well as normal mice on day 28 after tumor inoculation. CD45, Ly6G, Ly6C, CD11b and CXCR2 were analyzed. Compared the expression of Ly6G and Ly6C in Ly6G^+^Ly6C^+^ cells derived from normal mice, the phenotype of predominately expanded subset in tumor-bearing mice is Ly6G^mi^Ly6C^lo^. Ly6G^lo^Ly6C^hi^ subset was circled in green rectangle, Ly6G^hi^Ly6C^mi^ subset was in orange rectangle, and Ly6G^mi^Ly6C^lo^ subset was in red rectangle. Ly6G^mi^Ly6C^lo^CD11b^+^CXCR2^+^ subset was defined as CXCR2^+^ MDSCs. One representative image of 6 mice was shown. Percentage of CXCR2^+^ MDSCs in peripheral blood (**B**) and spleens (**C**) derived from normal mice or 4T1 tumor-bearing mice on day 0, 7, 14, 21, 28, and 35 after tumor inoculation was shown. ^*^P < 0.05, ^**^P < 0.01, ^***^P < 0.001. (**D**) Percentage of CXCR2^+^ MDSCs in peripheral blood, bone marrow (BM), spleens, tumor tissues, lungs and lymph nodes (LN) derived from 4T1 tumor-bearing mice on day 35 was shown.

MDSCs can be expanded and accumulated during tumor progression has been demonstrated *in vivo*. However, which subpopulation of MDSCs is predominantly expanding and recruiting remains unclear. Compared the expression of Ly6G and Ly6C in Ly6G^+^Ly6C^+^ cells derived from normal mice, CD45^+^Ly6G^mi^Ly6C^lo^CD11b^+^ subset was found to largely expand and accumulate in peripheral blood, spleens and tumor tissues in breast cancer mice model (Figure 1A). Notably, about 90% of these MDSC subsets in peripheral blood and 70% in spleens expressed CXCR2. Therefore, we defined these MDSC subsets as CXCR2^+^ MDSCs. Furthermore, the expansion of CXCR2^+^ MDSCs in peripheral blood and spleens was in a time-dependent manner during breast cancer progression (Figure 1B–1C). And CXCR2^+^ MDSCs exhibited a high proportion in peripheral blood, bone marrow, spleens and tumor tissues derived from every tumor-bearing mouse on day 35 after breast cancer cells inoculation (Figure 1D). However, unlike the similar percentage of CXCR2^+^ MDSCs in above mentioned organs and tissues, the percentage of CXCR2^+^ MDSCs was markedly different in lungs and axillary lymph nodes (Figure 1D). The discrepant proportion of CXCR2^+^ MDSCs was ranging from ∼30% to ∼2% and ∼30% to ∼0.2% in lungs and axillary lymph nodes respectively (Figure 1D). These results suggest that CXCR2^+^ MDSCs may be involved in tumor metastasis to lung or lymph node.

### CXCR2^+^ MDSCs are relevant to breast cancer metastasis to lung or lymph node

**Figure 2:**
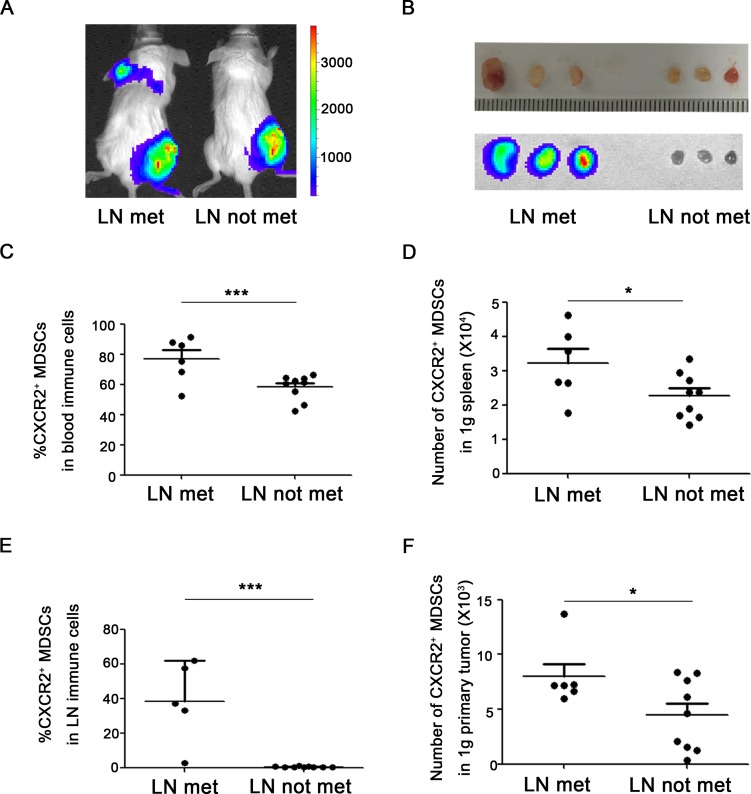
CXCR2^+^ MDSCs are relevant to breast cancer lymph node metastasis (**A**) Representative image of mice with or without axillary lymph node metastasis in 4T1 tumor-bearing mice using *in vivo* bioluminescence imaging. (**B**) Metastatic axillary lymph nodes were confirmed by bioluminescence imaging. (**C**) Percentage of CXCR2^+^ MDSCs in immune cells of peripheral blood from 4T1 tumor-bearing mice with or without axillary lymph node metastasis. (**D**) Number of CXCR2^+^ MDSCs in 1g spleen of 4T1 tumor-bearing mice with or without axillary lymph node metastasis. (**E**) Percentage of CXCR2^+^ MDSCs in immune cells of axillary lymph nodes from 4T1 tumor-bearing mice with or without axillary lymph node metastasis. (**F**) Number of CXCR2^+^ MDSCs in 1g primary tumor of 4T1 tumor-bearing mice with or without axillary lymph node metastasis. ^*^P < 0.05,^***^P < 0.001. Data was presented by mean ± sem.

**Figure 3:**
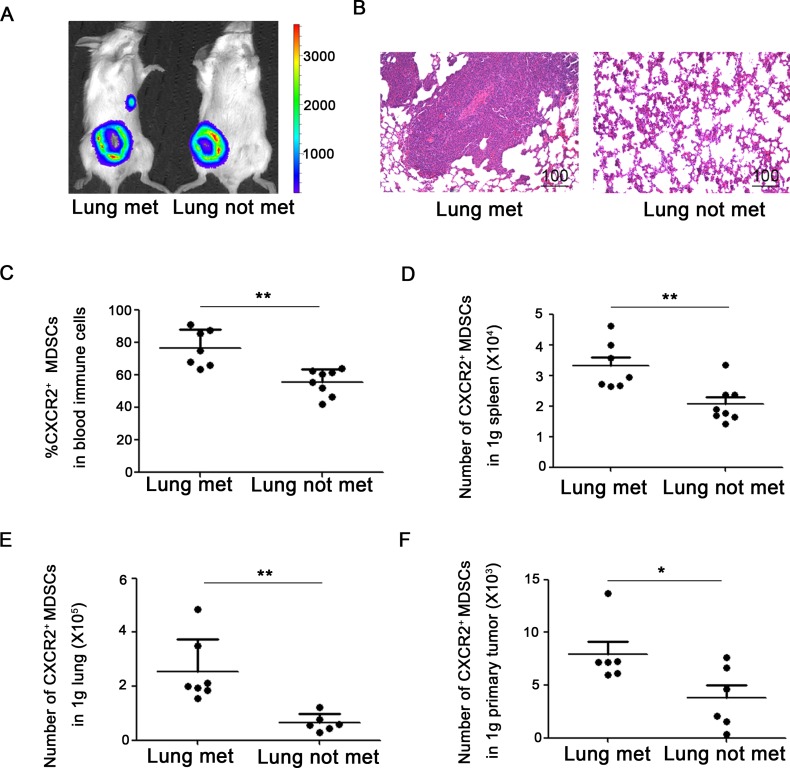
CXCR2^+^ MDSCs are involved in breast cancer lung metastasis (**A**) Representative image of mice with or without lung metastasis in 4T1 tumor-bearing mice using *in vivo* bioluminescence imaging. (**B**) Metastatic lung was confirmed by hematoxylin & eosin staining. (**C**) Percentage of CXCR2^+^ MDSCs in immune cells of peripheral blood from 4T1 tumor-bearing mice with or without lung metastasis. (**D**) Number of CXCR2^+^ MDSCs in 1g spleen of 4T1 tumor-bearing mice with or without lung metastasis. (**E**) Number of CXCR2^+^ MDSCs in 1g lung of mice 4T1 tumor-bearing with or without lung metastasis. (**F**) Number of CXCR2^+^ MDSCs in 1g primary tumor of 4T1 tumor-bearing mice with or without lung metastasis. ^*^P < 0.05, ^**^P < 0.01. Data was presented by mean ± sem.

To figure out the significance of the discrepant proportion of CXCR2^+^ MDSCs in lungs or lymph nodes, 4T1-luc cells were inoculated into mice, and the tumor-bearing mice were divided into different groups according to whether or not metastasis to lung or lymph node. Metastatic sites including lungs or axillary lymph nodes were confirmed by *in vivo* bioluminescence imaging and hematoxylin & eosin staining (Figure 2A–2B, Figure 3A–3B). Compared to mice without tumor metastatic, the percentage of CXCR2^+^ MDSCs in the peripheral blood was higher in mice with lymph node metastasis (76.7 ± 6.0% vs 58.1 ± 2.8%) (Figure 2C). Consistently, the number of CXCR2^+^ MDSCs in 1g spleen and 1g primary tumor was also higher in mice with lymph node metastatic (32104.2 ± 3888.7 *vs* 22673.2 ± 1950.3 in 1g spleen, 8000.0 ± 1059.4 vs 4476.6 ± 1016.4 in 1g primary tumor) (Figure 2D, 2F). More interestingly, the percentage of CXCR2^+^ MDSCs in metastatic lymph nodes was significantly higher than that in mice without lymph node metastatic (38.6 ± 10.5% vs 0.6 ± 0.1%) (Figure 2E). These results suggest that CXCR2^+^ MDSCs may be involved in tumor lymph node metastasis. Furthermore, in lung metastatic tumor model, we also found that compared to without lung metastatic mice, the percentage of CXCR2^+^ MDSCs in peripheral blood was higher in mice with lung metastasis (76.8 ± 4.0% *vs* 55.7 ± 3.0%), and the number of CXCR2^+^ MDSCs in 1g spleen and 1g primary tumor was also higher in mice with lung metastatic (33070.7 ± 2718.0 *vs* 20648.6 ± 1932.2 in 1g spleen, 7916.5 ± 1079.8 *vs* 3814.2 ± 1090.3 in 1g primary tumor), and the number of CXCR2^+^ MDSCs in 1g lung was higher in mice with lung metastatic mice (254317.1 ± 38711.1 vs 64163.3 ± 39316.9) (Figure 3C–3F). Taken together, the phenomena observed above implied that CXCR2^+^ MDSCs played a key role during breast cancer metastasis to lung or lymph node.

### CXCR2^+^ MDSCs promote breast cancer growth and metastasis *in vitro* and *in vivo*

**Figure 4:**
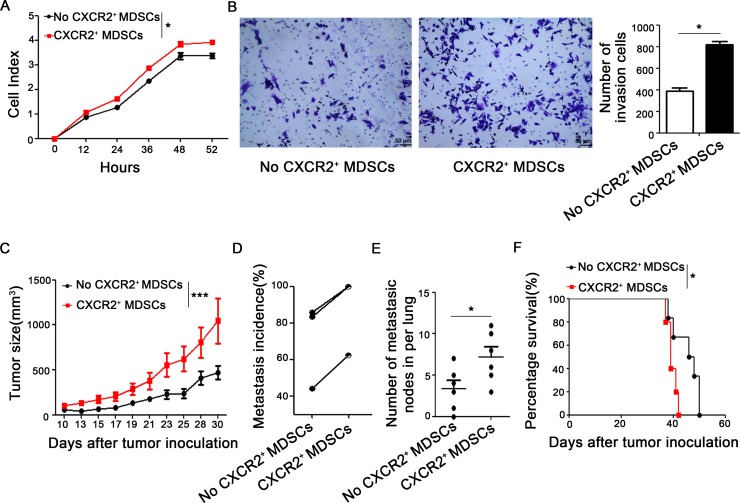
CXCR2^+^ MDSCs promote breast cancer progression (**A**) Proliferation of 4T1 cells co-cultured with or without CXCR2^+^ MDSCs (4T1:CXCR2^+^ MDSCs 1:5) was supervised by Real-Time Cell Analyzing (RTCA) methods. (**B**) Invasion ability of 4T1 cells which interplayed with or without CXCR2^+^ MDSCs (4T1:CXCR2^+^ MDSCs 1:5) was recorded. (**C**) Tumor size of 4T1 tumor-bearing mice which was injected with or without CXCR2^+^ MDSCs (4T1:CXCR2^+^MDSC 1:50) was measured by vernier caliper and recorded. (**D**) Metastasis incidence- either lung or lymph node metastasis- of three experiments of 4T1 tumor-bearing mice with or without CXCR2^+^ MDSCs inoculation (4T1:CXCR2^+^MDSC 1:50). Each line connected metastatic data from two groups of respective experiment and there were at least 6 mice per group. (**E**) Number of metastatic nodes in lungs from 4T1 with or without CXCR2^+^ MDSCs inoculation mice (4T1:CXCR2^+^MDSC 1:50). (**F**) Representative survival curve of 4T1 with or without CXCR2^+^ MDSCs inoculation mice (4T1:CXCR2^+^MDSC 1:50). There were 5 and 6 mice in 4T1 with or without CXCR2^+^ MDSCs inoculation group respectively. ^*^P < 0.05, ^**^P < 0.01. Data was presented by mean ± sem. Every experiment was replicated at least three times.

Next, we want to explore whether CXCR2^+^ MDSCs promote breast cancer progression and metastasis *in vitro* and *in vivo*. 4T1 cells were co-cultured with or without CXCR2^+^ MDSCs sorted from spleen of 4T1-tumor bearing mice, *in vitro* proliferation was supervised by Real-Time Cell Analyzing (RTCA, AceaBio, China) method. The results shown in Figure 4A, 4T1 cells co-cultured with CXCR2^+^ MDSCs presented higher cell index and showed greater capability of proliferation than those without CXCR2^+^ MDSCs. Moreover, the invasion ability of 4T1 cells co-cultured with CXCR2^+^ MDSCs was also significantly higher than that of without CXCR2^+^ MDSCs (Figure 4B). Consistently, the growth of 4T1 tumors which were injected together with CXCR2^+^ MDSCs sorted from spleen derived from mice was faster than those without CXCR2^+^ MDSCs injection *in vivo* (Figure 4C). Furthermore, CXCR2^+^ MDSCs could significantly aggravated tumor metastasis (Figure 4D). The axillary lymph nodes from the mice injected 4T1 together with CXCR2^+^ MDSCs exhibited larger sizes and more metastatic nodes (66.7% vs 33.3%, confirmed by hematoxylin & eosin staining, data not shown) than those without CXCR2^+^ MDSCs injection. The lungs from the mice injected 4T1 together with CXCR2^+^ MDSCs showed more metastatic sites (Figure 4E). Moreover, the life length of mice was shortened when injected 4T1 together with CXCR2^+^ MDSCs (Figure 4F). Collectively, these results demonstrated that CXCR2^+^ MDSCs could promote breast cancer progression and metastasis.

### CXCR2^+^ MDSCs induce breast cancer cell EMT via IL-6

**Figure 5:**
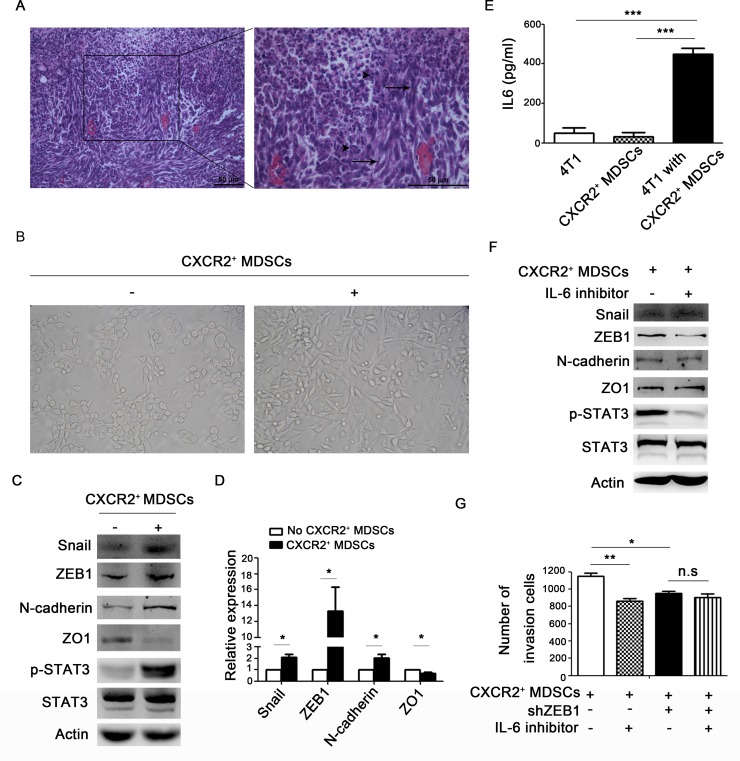
CXCR2^+^ MDSCs induce breast cancer cells EMT (**A**) Representative image of metastatic axillary lymph node by hematoxylin & eosin (HE) staining. Black arrows pointed to spindle tumor cells. Arrow head pointed to granulocytes. (**B**–**G**) 4T1 cells were co-cultured with or without CXCR2^+^ MDSCs (4T1:CXCR2^+^ MDSCs 1:5), image of the morphology of 4T1 cells in light field (B). 4T1 cells were co-cultured with or without CXCR2^+^ MDSCs for 42h, and protein expression of 4T1 cells was detected by western-blot (C). mRNA expression of 4T1 cells co-cultured with or without CXCR2^+^ MDSCs was detected by quantitative PCR (D). IL-6 concentration in the supernatant of 4T1 culture medium alone, CXCR2^+^ MDSCs culture medium alone and 4T1 and CXCR2^+^ MDSCs co-culture medium was detected by ELISA (E). Protein expression of 4T1 cells treated with or without IL-6 inhibitor when co-cultured with CXCR2^+^ MDSCs, was measured by western-blot (F). Invasion ability of 4T1 cells and 4T1-shZEB1 cells, treated with or without IL-6 inhibitor when co-cultured with CXCR2^+^ MDSCs was recorded (G). ^*^P < 0.05, ^***^P < 0.001. Data was presented by mean ± sem. Every experiment was replicated at least three times.

In metastatic lymph nodes, we found that the tumor cells around infiltrated MDSCs were usually in spindle shapes (Figure 5A). Then, whether CXCR2^+^ MDSCs could promote breast cancer cells EMT needed to be investigated. First, 4T1 cells co-cultured with CXCR2^+^ MDSCs sorted from spleen of tumor-bearing mice displayed mesenchymal-like morphology *in vitro* (Figure 5B). Next, 4T1 cells co-cultured with CXCR2^+^ MDSCs reduced the epithelial marker ZO1 expression, but increased the expression of mesenchymal marker ZEB1, Snail and N-cadherin (Figure 5C–5D). Moreover, when 4T1 cells were co-cultured with CXCR2^+^ MDSCs, p-STAT3 was profoundly enhanced (Figure 5C). p-STAT3 is mainly the downstream of IL-6 signaling pathway, and IL-6 concentration in the supernatant of 4T1/CXCR2^+^ MDSCs co-culture system was significantly higher than that in 4T1 cells or CXCR2^+^ MDSCs cultured alone (Figure 5E). These data demonstrated that IL-6 might participate in EMT occurrence of 4T1 cells when co-cultured with CXCR2^+^ MDSCs. Moreover, blockade of IL-6 signaling could markedly reduce ZEB1 expression and impair the invasive ability of 4T1 cells co-cultured with CXCR2^+^ MDSCs in the co-culture system (Figure 5F–5G). Additionally, when knocking down the expression of ZEB1 in 4T1 cells, the invasive ability of cancer cells was similar with IL-6 signal inhibition (Figure 5G). These above observation demonstrated that CXCR2^+^ MDSCs could promote cancer cells EMT via IL-6.

### CXCR2^+^ MDSCs induce activated T cell exhaustion partially via IFN-γ

**Figure 6:**
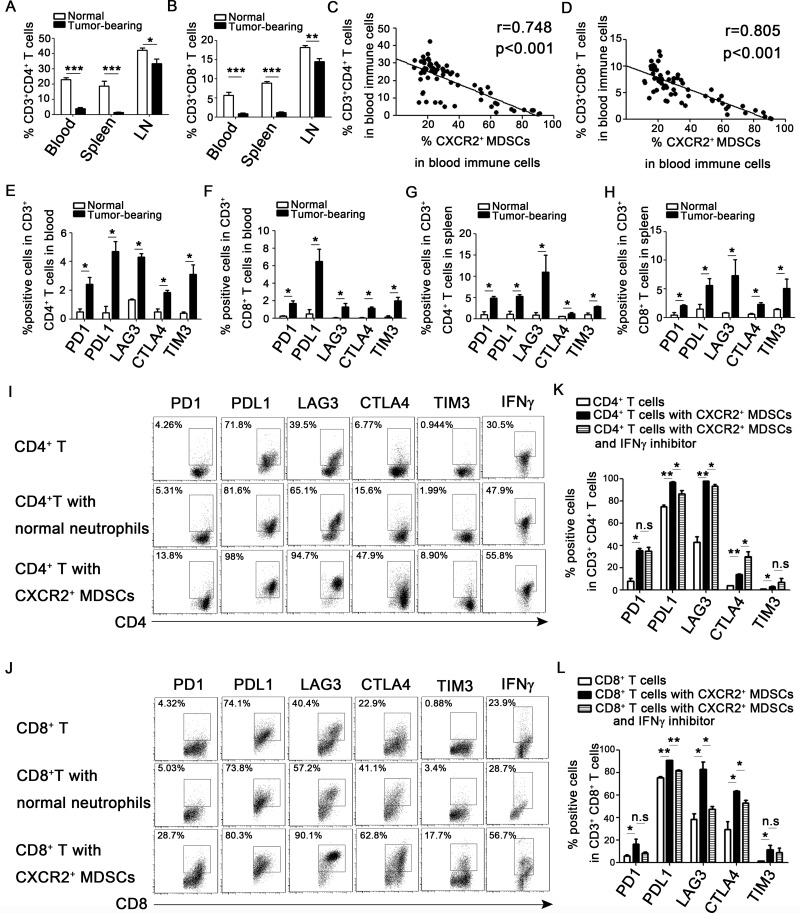
CXCR2^+^ MDSCs induce activated T cells exhaustion (**A**–**D**) 4T1 tumor-bearing mice were sacrificed on day 0, 7, 14, 21, 28, 35 after subcutaneously injected with 4 × 10^5^ 4T1 cells. Single cells of peripheral blood, spleen and axillary lymph nodes (LN) derived from 4T1 tumor-bearing or normal mice were obtained, and analyzed by flow cytometric staining with CD3, CD4, CD8, Ly6G, Ly6C, CD11b, and CXCR2 antibody as described in the Methods. (A) Percentage of CD3^+^CD4^+^ T cells in immune cells of peripheral blood, spleen and axillary lymph nodes on day 35 after tumor injection. (B) Percentage of CD3^+^CD8^+^ T cells in immune cells of peripheral blood, spleens and axillary lymph nodes on day 35 after tumor inoculation. (C, D) Correlation analysis between the percentage of CD3^+^CD4^+^ T cells (C) or CD3^+^CD8^+^ T cells (D) and CXCR2^+^ MDSCs. (**E**–**H**) The checkpoint molecular expression on CD3^+^CD4^+^ and CD3^+^CD8^+^ T cells in peripheral blood and spleen derived from normal or tumor-bearing mice. (**I**, **J**) The expression of checkpoint molecular and IFN-γ of CD3^+^CD4^+^ and CD3^+^CD8^+^ T cells cultured with or without CXCR2^−^ cells from normal mice (T cells: CXCR2^−^cells 1:1) or CXCR2^+^ MDSCs from tumor-bearing mice (T cells: CXCR2^+^MDSCs 1:1). (**K**, **L**) The expression of checkpoint molecular of CD3^+^CD4^+^ or CD3^+^CD8^+^ T cells co-cultured with CXCR2^+^ MDSCs with or without IFNγ inhibitor. ^*^P < 0.05, ^**^P < 0.01, ^***^P < 0.001. Each experiment was replicated at least three times.

During the breast cancer progression, the proportion of CD3^+^CD4^+^ T cells and CD3^+^CD8^+^ T cells in peripheral blood, spleens and axillary lymph nodes were significantly decreased (Figure 6A–6B), and the decreased proportion of CD3^+^CD4^+^ T cells and CD3^+^CD8^+^ T cells were negatively relative to the increased CXCR2^+^ MDSCs in peripheral blood (Figure 6C–6D). Moreover, CD3^+^CD4^+^ T cells and CD3^+^CD8^+^ T cells derived from 4T1-tumor bearing mice expressed higher level of immunosuppressive markers including PD1, PDL1, LAG3, CTLA4 and TIM3 in peripheral blood and spleen than that in normal mice (Figure 6E–6H). And the expression of these immunosuppressive markers was also high in CD3^+^CD4^+^ T cells and CD3^+^CD8^+^ T cells in tumor tissues (data not shown). It was well known that MDSCs could suppress T cells function. So, we next investigated how CXCR2^+^ MDSCs affected T cells function *in vitro*. CD4^+^ or CD8^+^ T cells were cultured with or without Ly6G^+^CXCR2^−^ cells sorted from spleen of normal mice or CXCR2^+^ MDSCs sorted from spleen of 4T1 tumor-bearing mice under the stimulation of anti-CD3 and anti-CD28 for 3 days. The results shown in Figure 6I–6J, compared to those cultured alone or co-cultured with Ly6G^+^CXCR2^−^ cells, the immunosuppressive molecules including PD1, PDL1, LAG3, CTLA4, and TIM3 were significantly upregulated in both CD4^+^ T cells and CD8^+^ T cells when co-cultured with sorted CXCR2^+^ MDSCs. Additionally, the secretion of IFN-γ of CD4^+^ T cells and CD8^+^ T cells was increased when co-cultured with CXCR2^+^ MDSCs, while the secretion of Granzyme B, proferin and IL-2 did not show significant difference (data not shown). Moreover, when IFN-γ inhibitor was added to the co-culture system of T cells and CXCR2^+^ MDSCs, the results shown in Figure 6K–6L, blockade of IFN-γ signaling significantly reduced the expression of PDL1, LAG3 and CTLA4 in CD8^+^ T cells, also reduced the expression of PDL1 and LAG3 in CD4^+^ T cells, but increased the expression of CTLA4 in CD4^+^ T cells, didn't affect the PD1 and TIM3 expression in both CD4^+^ T cells and CD8^+^ T cells. The above results demonstrated that CXCR2^+^ MDSCs induced T cells exhaustion via IFN-γ partially. However, which factor(s) affected PD1 and TIM3 expression and why blockade of IFN-γ signaling increased CTLA4 expression in CD4^+^ T cells needed to be further investigated.

## DISCUSSION

It is well-known that metastatic breast cancer is incurable after the diagnosis of stage IV breast cancer, only 23% of patients can survive for 5 years [26]. The high level of MDSCs is related to poor prognosis of breast cancer and plays a key role in breast cancer progression [4]. MDSC is originally described as CD11b^+^Gr1^+^ cells, and Gr1 antigens Ly6G and Ly6C distinguish G-MDSCs and M-MDSCs respectively. Despite the advanced development on MDSC research, more detailed subsets are still in need for clinical application. Here, we identify that CD45^+^Ly6G^mi^Ly6C^lo^CD11b^+^ subpopulation are predominately expanded and recruited in systemic and local tumor microenvironment during breast cancer progression and metastasis, which expresses CXCR2 at high proportion, then we named this subset CXCR2^+^ MDSCs. Why Ly6G and Ly6C are downregulated in CXCR2^+^MDSCs during breast cancer progression need to be further investigated. In this study, we focus on the function of CXCR2^+^ MDSCs in breast cancer progression.

CXCL1/2, which can recruit Gr1^+^CD11b^+^ myeloid cells into tumor bed, has been reported to be highly expressed in metastatic breast cancer. CXCL1/2-CXCR2 axis may involve in breast cancer progression, and Ly6G^+^CD11b^+^ cells sorted from tumor tissues express higher levels of CXCR2 than Ly6C^+^CD11b^+^ cells, F4/80^+^ cells and CD31^+^ cells [27]. CXCR2 is required for homing Ly6G^hi^CD11b^+^ MDSCs from the circulatory system to tumor tissues in colitis-associated tumorigenesis. Consistently, CXCR2 is essential for Ly6G^hi^CD11b^+^ MDSCs trafficking into tumor tissues, but isn't required for these subsets to egress from the bone marrow in rhabdomyosarcoma. In *Cxcr2*^−/−^ mice, there are more accumulation of Ly6G^hi^CD11b^+^ MDSCs in peripheral blood, while reducing recruitment of these MDSCs subsets in local tumor tissues results in retarding tumor progression. Moreover, loss of CXCR2 in MDSCs can enhance CD8^+^ T cells cytotoxicity against tumor cells without affecting the number of CD8^+^ T cells [18]. Additionally, blocking CXCR2 in Ly6G^hi^CD11b^+^ MDSCs significantly enhances PD1 checkpoint blockade efficacy *in vivo* [19]. Furthermore, Gr1^hi^CD11b^hi^CXCR2^+^ MDSCs could enhance T cell lymphoma growth by promoting angiogenesis, and boost lung cancer or mesothelioma via pro-angiogenesis and immunosuppression on T cells [28]. These above studies addressed the function of CXCR2 on MDSCs recruitment from periphery to tumor tissues, and demonstrated predominate local pro-tumor effects of Ly6G^+^CD11b^+^CXCR2^+^ MDSCs in tumor tissues. Here, we confirmed that Ly6G^mi^Ly6C^lo^CD11b^+^CXCR2^+^ MDSC subsets (CXCR2^+^ MDSCs) were the main subpopulation of MDSCs expanding and recruiting during breast cancer progression. The proportion and numbers of CXCR2^+^ MDSCs markedly increased in peripheral blood, bone marrow, spleen, primary tumor tissues, especially in tumor metastatic lung or lymph nodes, and delineated that CXCR2^+^ MDSCs not only accelerated tumor growth, but also promoted tumor metastasis to lung or lymph nodes.

It has been demonstrated that MDSCs implicate in reshaping breast cancer cells and endow breast cancer cells with stem cell-like qualities [29]. Stem-like cancer cells play an essential role in metastatic initiation, and MDSCs in metastatic sites will enlarge the stem cell-like cancer cells pool [30]. Here, we found that CXCR2^+^ MDSCs could reshape breast cancer cells to mesenchymal-like via boosting EMT. EMT is believed to be a critical step in metastatic process. Changes in EMT regulatory pathways lead to loss of cellular adhesion, changes of cell polarization, migration, intra- or extra-vasation and finally metastasis [31]. After co-culture with CXCR2^+^ MDSCs system, breast cancer cells exhibited mesenchymal-like morphology, the expression of ZEB1, Snail and N-cadherin was up-regulated, and ZO1 was down-regulated. ZEB1, Snail and N-cadherin are markers for mesenchymal phenotype, while ZO1 is for epithelial phenotype. However, which factor is involved in this EMT process? A growing list of EMT regulators has been identified, including TGF-β, IL-6, HGF, FGF, IGF and Notch ligands [32]. It has been demonstrated that high levels of IL-6 have been detected in serum of patients suffering from breast cancer [33]. IL-6/STAT3 signaling can effectively trigger EMT action and expand the cancer stem cells population in several types of tumors [34, 35]. Moreover, MDSCs expanding are positively correlated with the elevated serum IL-6 levels in nasopharyngeal carcinoma. MDSCs promote nasopharyngeal cancer cells migration and invasion by triggering the EMT via cell-to-cell contact, and MDSCs enhance tumor experimental lung metastasis *in vivo* [36]. Additionally, MDSCs can induce angiogenesis in a STAT3 dependent manner [37]. Therefore, we detected IL-6 secretion in the supernatant of co-culture medium of breast cancer cells and CXCR2^+^ MDSCs. To our surprise, there was dramatically higher level of IL-6 in the co-culture medium. However, whether CXCR2^+^ MDSCs-induced 4T1 cells EMT was IL-6 dependent? Then, IL-6 inhibitor was used to block IL-6/STAT3 signaling, and found that could significantly rescue the expression of ZEB1, Snail and N-cadherin but not Snail and N-cadherin, and hamper the invasion ability of 4T1 cells. Furthermore, knocking down ZEB1 expression in 4T1 cells, IL-6 inhibitor didn't significantly hamper 4T1 cells invasion activation. Similar to our results, it has been reported that stimulation with IL-6 will up-regulate the expression of ZEB1 and down-regulate ZO1 expression in colon cancer cells [38]. These results demonstrated that IL-6 was a key factor in CXCR2^+^ MDSCs mediated breast cancer cells EMT.

A major breakthrough of cancer immunotherapy is the discovery of T cell checkpoint blockade pathways. Several clinical trials of PD1/PDL1 blockade, alone or combination with CTLA4 therapy are ongoing in advanced triple negative breast cancer [39]. Rescuing the exhausted states of T cells during tumor progression is an important mechanism for checkpoint blockade anti-tumor strategy [40]. Exhausted T cells express high levels of inhibitory receptors, including PD1, PDL1, LAG3, CTLA4, and TIM3 [41], which experience loss of proliferation, cytokine production and cytotoxic activity [42]. PD1 is expressed on T cells following T cell receptor (TCR) activation. PDL1 is abundantly expressed in cancer cells and stromal cells. Blockade of PDL1/PD1 dampens T cell anergy and apoptosis, thus enhancing antitumor immune responses [41, 43]. LAG3 expresses on activated CD4^+^ and CD8^+^ T cells, which can negatively regulate T cell expansion by inhibiting TCR-induced calcium fluxes, resulting in controlling the size of the memory T cell pool. Moreover, LAG3 signaling contributes to CD4^+^ regulatory T cell suppression of autoimmune responses and CD8^+^ T cells tolerance to self and tumor antigens. TIM3 is a member of TIM family and is expressed by T helper 1 cells (Th1), DCs, CD8^+^ T cells and other lymphocyte subsets. Interaction of TIM3 and its ligand galectin 9 can inhibit T cell proliferation and cytokine secretion [43]. CTLA4 is only expressed in T cells, which competes with the co-stimulatory molecule CD28 in binding the ligands CD80/CD86 and initiating intracellular inhibitory signals [41]. It has been reported that the severity of T cells exhaustion is determined by the strength of inhibitory receptors expression [44]. The presence of PD1^high^ exhausted T-cells within tumor microenvironments were associated with resistance to anti-PD1 therapy. However, exhausted T-cells with either PD1^low^ or PD1^int^ expression retain their capacity to be reinvigorated by anti-PD1 treatment [45]. It has been reported that infiltrating MDSCs could induce PD1 expression on CD4^+^ effector memory T cells in glioma [46]. Our study declared that CXCR2^+^ MDSCs markedly enhanced the expression of PD1, PDL1, LAG3, CTLA4 and TIM3 and increased IFN-γ secretion in activated CD4^+^ or CD8^+^ T cells. Fu *et al*. have shown that neutralization of IFN-γ can reduce the expression of PDL1 in tumor microenvironment [47]. So, we added IFN-γ inhibitor in the T cells and CXCR2^+^ MDSCs co-culture system, found that IFN-γ not only regulated the expression of PDL1, but also regulated the expression of LAG3 and CTLA4. And the function of IFN-γ was more robust in CD8^+^ T cells than in CD4^+^ T cells. Therefore, our data expanded the knowledge about MDSCs and T cell exhaustion in breast cancer. However, there were a few limitations in our present study. Though the function of CXCR2 in tumor progression has been widely investigated and reported to participate in angiogenesis [48], chemo-resistance [49], EMT [49] and anti-PD1 treatment efficiencyb [19], the precise function and mechanism of CXCR2 in tumor metastasis to lung or lymph node of CXCR2^+^ MDSCs need to be further investigation.

In summary, here we identified a subpopulation of MDSCs in breast cancer which phenotype is Ly6G^mi^Ly6C^lo^CD11b^+^CXCR2^+^ (CXCR2^+^ MDSCs). CXCR2^+^ MDSCs could expand and recruit during breast cancer progression, and promoted primary cancer cells metastasize to lung or lymph node. Moreover, CXCR2^+^ MDSCs could induce breast cancer cells EMT via IL-6, and promote activated CD4^+^ or CD8^+^ T cells exhaustion partially via IFN-γ. These results suggest that CXCR2^+^ MDSCs subsets may be a potential candidate therapeutic target of breast cancer.

## MATERIALS AND METHODS

### Mice and cell lines

Female BALB/c mice (6–8 weeks) were obtained from Joint Ventures Sipper BK Experimental Animal (Shanghai, China). All animal experiments were undertaken in accordance with the National Institute of Health Guide for the Care and Use of Laboratory Animals, with the approval of the Scientific Investigation Board of Second Military Medical University, Shanghai, China. The 4T1 mammary carcinoma cell line derived from BALB/c origin were obtained from the American Type Culture Collection (Manassas, VA, USA) and maintained in RPMI1640 complete medium (PAA Laboratories, Linz, Austria) supplemented with 10% (vol/vol) fetal bovine serum (FBS, PAA Laboratories) at 37°C in 5% CO2 atmosphere. Luciferase-labeled 4T1 cells (4T1-luc cells) were obtained from PerkinElmer Inc (Hopkinton, MA, USA) and maintained in RPMI 1640 containing 10% FBS and G418 (200 ng/ml, Sigma). 4T1 was transfected with GFP expressing shRNA plasmid for ZEB1 (designed by OriGene) by JetPEI (Polyplus-transfection, France) and sorted by FACS sorting system (Sony) into 96 well plate with the density of one cell per well. The expression of ZEB1 in transfected cell clones was analyzed by real-time PCR and the stably silenced transfected cell clones were maintained in RPMI 1640 containing 10% FBS and 1ug/ml purine.

### Reagents

ELISA kits for murine IL-6 was from R&D Systems (Minneapolis, MN). Fluorescein-conjugated mAbs including anti-CD3-PE-Cy7, CD4-PE-Cy5, CD8-PE, CD8-FITC, CD11b-APC, Ly6G-PE, Ly6C-FITC, CXCR2-Percp-Cy5.5, CD45-BV510, PD1-PE, PDL1-PE, LAG3-PE, CTLA4-PE, IFNγ-PE and isotype antibodies were purchased from BD biosciences. TIM3-PE was from Miltenyi Biotec (Bergisch-Gladbach, Germany). Antibody against STAT3 (79D7, 4904S), p-STAT3 (Tyr705, 9131S), ZEB1 (3396P), ZO-1(5406P), Snail(3879P), N-Cadherin(4061P) were obtained from Cell Signaling Technology (Beverly, MA, USA) and antibody against Actin was from Santa Cruz. IL6 inhibitor (501109) and IFN-γ inhibitor (505827) was from Biolegend. Luciferin substrate (K9909PE) for *in vivo* image was purchased from PerkinElmer Inc (Hopkinton, MA, USA). HE staining kit was from Beyotime Biotechnology. ShRNA for ZEB1 (TG513177) was purchased from OriGene.

### Preparation and observation of tumor-bearing mice

4 × 10^5^ 4T1 or 4T1-luc cells suspended in 100ul PBS were subcutaneously into the right flank of the fourth mammary gland of Balb/c mice. The tumor sizes for 4T1 tumor-bearing mice were measured with a caliper after tumor inoculation every 2∼3 days and the tumor volumes were determined by measuring of the maximal (a) and minimal (b) diameters and calculated by using the formula a × b^2^/2. The tumor growth for 4T1-luc tumor-bearing mice was evaluated by bioluminescence imaging signal. 4T1-luc tumor-bearing mice were intraperitoneally injected with 100ul luciferin substrate (5 mg/ml) for 15 minutes, and then anesthetized by isoflurane before taken photogragh by IVIS Lumina K series III (PerkinElmer Inc, Hopkinton, MA, USA), and all images were adjusted to the same exposure time. The survival of the tumor-bearing mice was monitored daily. In some experiments, mice were sacrificed on day 35 after tumor inoculation, the lungs and axillary lymph nodes were photographed and resected, fixed in 4% paraformaldehyde, embedded in paraffin and sectioned. The sections were stained with hematoxylin and eosin (H&E). For some experiments, the lungs were perfused with ink which was mixed with 4% paraformaldehyde in a ratio of 1:1, and then were de-colored by ink de-coloring liquid (mixed liquid (vol/vol) with 70% ethanol, 10% methanol, 5% glacial acetic acid and 15% double distilled water) for half an hour. Experiments were performed independently three times, and each group contained 6 mice at least.

### CXCR2^+^ MDSCs isolation

Single cell suspension from spleen of tumor bearing mice was stained with Ly6G-PE, Ly6C-FITC, CD11b-APC and CXCR2-Percp-cy5.5, then the Ly6G^mi^Ly6C^lo^CD11b^+^CXCR2^+^ subpopulations (CXCR2^+^ MDSCs) were sorted by a MoFlo XDP flow cytometer (Beckman-Coulter) with purities of >95%. 2 × 10^5^/ml 4T1 cells and 1 × 10^6^/ml isolated CXCR2^+^ MDSCs (1:5) were seeded into 6 well flat-bottom plates for co-culture system. On day 7 after 4 × 10^5^ 4T1 cells were inoculation into BALB/c mice, 2 × 10^7^ isolated CXCR2^+^ MDSCs (1:50) were intra-tumorally injected in 50ul PBS, the tumor growth and survival of tumor-bearing mice were measured as described above.

### Flow cytometry analysis

Single-cell suspension of spleens, axillary lymph nodes, bone marrow, peripheral blood, lung and tumor tissues was prepared as described previously [50]. For flow cytometry, 1 × 10^6^ cells were labeled for flow cytometric analysis with a FACS LSRII (BD Biosciences) and data were analyzed with FACS Diva software. PE-Cy7, PE-Cy5, PE, FITC, APC, PerCP-Cy5.5, or BV510 Abs were used to recognize CD3, CD4, CD8, Ly6G, Ly6C, CD11b, CXCR2, CD45, PD1, PDL1, LAG3, CTLA4 and TIM3. Intracellular staining was performed according to the instructions of BD Cytofix/Cytoperm^™^ Plus kit (5123796).

### Western-blot analysis

Western blot was performed as previously described [51]. Briefly, cells were lysed and protein concentration was determined by the BCA Protein Assay kit (Pierce, Rockford, IL, USA). Cell lysates were separated by SDS-PAGE gels and transferred to nitrocellulose membranes. Membranes were blotted with the indicated antibodies. Proteins were visualized using SuperSignal West Femto Maximum Sensitivity Substrate, as instructed by the manufacturer (Pierce, Rockford, IL, USA).

### Reverse-transcription PCR and real-time PCR

Total cellular RNA was extracted with Trizol reagent (Invitrogen) according to manufacturer’s instructions. 600 ng of total RNA was used in a 10 ul reverse-transcription reaction using Rever Tra Ace qPCR RT Kit (FSQ-101, Toyobo). A light Cycler (Roche) and a SYBR RT-PCR kit (Takara) were used for quantitative real-time PCR analysis according to the manufacturer’s protocol. Primer sequences used for PCR amplification were 5′-GCCGCTAAGAGCACAGCAA-3′ and 5′-TCCCCAC

TCTGAAAATGAGGA-3′ for ZO-1, 5′-GCTGGCAAGA

CAACGTGAAAG-3′ and 5′-GCCTCAGGATAAATGAC GGC-3′ for ZEB1, 5′-AGTGTGACGTTGACATCCGT-3′ and 5′ GCAGCTCAGTAACAGTCCGC-3′ for Actin.

### Invasion assay

The invasion of tumor cells was evaluated by the Matrigel (10 μm thickness and 8 μm pore size) assay using 24-well Boyden chambers (BD Biosciences, Bedford, MA, USA) containing a polycarbonate membrane according to the manual. 1 × 10^5^ cells in 200 μl RPMI 1640 medium were added to the upper compartment of chamber. 5 × 10^5^ isolated CXCR2^+^ MDSCs in 600μl RPMI 1640 medium with 10% (vol/vol) fetal bovine serum were placed in the bottom chamber. When testing the function of IL-6, 5 ug/ml IL-6 inhibitor was added to both upper and bottom chamber. After incubation for 24 hours, filters were harvested, fixed in 4% paraformaldehyde for 30 minutes and stained with 0.5% crystal violet (Sangon) for 2 minutes. Cells in the upper chamber were removed with cotton swabs, and the number of invaded cells was counted under a microscope in 10 pre-determined fields at 200-fold magnification. Results were presented as mean ± sem.

### Enzyme-linked immunosorbent assay (ELISA)

Supernatant of culture medium was centrifuged 1000 rpm for 5 minutes to get rid of cells. Protein levels of the prepared supernatant were measured by mouse Quantikine ELISA kits (R&D) according to the manufacturer’s protocols.

### Co-culture system

CXCR2^+^ MDSCs from spleens of 4T1 tumor-bearing mice, and CXCR2^−^ cells from normal mice were sorted by a MoFlo XDP flow cytometer (Beckman-Coulter) with purities of >95% as described above. Then they were co-cultured with CD4^+^ T cells (5 × 10^4^) or CD8^+^ T cells (1 × 10^5^) respectively at 1:1 ratio in the presence of 2.5 ug/ml anti-mouse CD3 and 1.25 ug/ml anti-mouse CD28. After 3 days incubation, the expression of PD1, PDL1, LAG3, CTLA4, TIM3, IFN-γ, Perforin, Granzyme B, and IL-2 in CD4^+^ T or CD8^+^ T cells were detected by flow cytometry analysis. The co-cultured system was added with 10ug/ml IFN-γ inhibitor to test the function of IFN-γ.

### Statistical analysis

All experiments were performed independently at least three times. Results were provided as the mean ± sem. Comparison of mean values between groups was determined by unpaired Student’s *t*-test. Statistical analysis of survival data was performed by the Kaplan–Meier method and analyzed by the log-rank test. *P* values < 0.05 was considered to be statistically significant. All statistics were analyzed with the assistance of Graphpad Prism 5.0. Correlation analysis was conducted using SPSS 19.0 with *r* values and *p* values shown.
